# Dr. Howard A. Kelly's *The Stereo Clinic*: health science pedagogy and the egalitarian future of 3D clinical visualization

**DOI:** 10.5195/jmla.2022.1450

**Published:** 2022-04-01

**Authors:** Sebastian C. Galbo, Keith C. Mages

**Affiliations:** 1 scgalbo@buffalo.edu, PhD Student, University at Buffalo Department of English; Graduate Assistant, Robert L. Brown History of Medicine Collection, University Libraries, Buffalo, NY; 2 kcmages@buffalo.edu, Curator, Robert L. Brown History of Medicine Collection; Associate Librarian, University Libraries, University at Buffalo, Buffalo, NY

**Keywords:** health sciences education, pedagogy, 3D, history of medicine, stereoscope, stereoscopic imagery

## Abstract

This article situates emerging three-dimensional (3D) visualization technologies in the health sciences within the broader historical context of the stereoscope. Although 3D visualization technologies enhance pedagogy and deepen student engagement, they are generally cost-prohibitive and therefore inaccessible for many institutions. In light of this issue, the authors consider the work of American gynecologist and founding member of The Johns Hopkins Hospital in Baltimore, Maryland, Dr. Howard Atwood Kelly (1858–1943). A monumental work, Kelly's *The Stereo Clinic* is a multivolume publication whose focal point was the stereoscope, an image-viewing device that can be seen as a prototype for present-day 3D technologies. Each installment presents a step-by-step overview of a specific surgical procedure using a didactic narrative and corresponding stereoscopic images that illustrate the clinical practices. Significantly, Kelly understood *The Stereo Clinic* as an egalitarian project that provided high-quality educational resources to students and practicing physicians who did not have access to world-class clinical suites and teaching institutions. Furthermore, he viewed *The Stereo Clinic* as a remedy to the commonplace frustrations of medical education, such as crowded surgical suites, and the hazards of in-person observation. *The Stereo Clinic* is an important case study because it reveals a medical profession at the turn of the twentieth century preoccupied with 3D visualization. Inventive clinicians such as Kelly did not only advocate for this technology on the strength of its pedagogical value; they also articulated the equalitarian nature of this medium and produced 3D technology accessible to a wide audience.

## THE STEREOSCOPE: FROM RECREATIONAL COMMODITY TO VALUED EDUCATIONAL TOOL

Three-dimensional (3D) visualization of the human body is an area of active interest and investigation among the health sciences community. Recent scholarship shows that 3D imagery enhances medical student enthusiasm while augmenting anatomic knowledge acquisition and spatial visualization [1, 2]. Among surgical trainees, 3D visualization correlates with increased awareness of anatomical details, including “distances and orientations between microstructures” [3]. Likewise, when compared with traditional two-dimensional (2D) video instruction of surgical procedures, 3D viewing enables either comparative or increased comprehension of anatomical structures [4]. Although beneficial, novel 3D virtual reality (VR) technologies may be cost-prohibitive. On this, physician Jack Pottle notes: “Virtual simulation costs often comprises hardware and software. High-end VR hardware costs approximately £3,000 [$4,100] for a setup (laptop and headset)” [5].

Health sciences libraries have emerged as important spaces to connect users with often cost-prohibitive 3D hardware and software [6]. Likewise, librarians are at the forefront of pedagogic technologies, functioning as early adopters, evaluators, and champions [7, 8]. Due to their unique role, we encourage the library community to take a closer look at Dr. Howard Kelly's fascinating, yet largely overlooked, multivolume set, *The Stereo Clinic* [9–20].

Published in installments from 1908 to 1915, this work included hundreds of stereoviews that, when viewed on a stereoscope, depicted various diagnostic and surgical procedures in vivid 3D. *The Stereo Clinic* is an important case study as it reveals a medical profession at the turn of the twentieth-century preoccupied with 3D visualization. The present-day enthusiasm for this technology, then, should not be understood as wholly novel, but instead as an extension of this earlier era. Inventive clinicians such as Kelly, however, did not only advocate for this technology on the strength of its pedagogical value; they also discerned and articulated the equalitarian nature of this medium.

**Figure 1:**
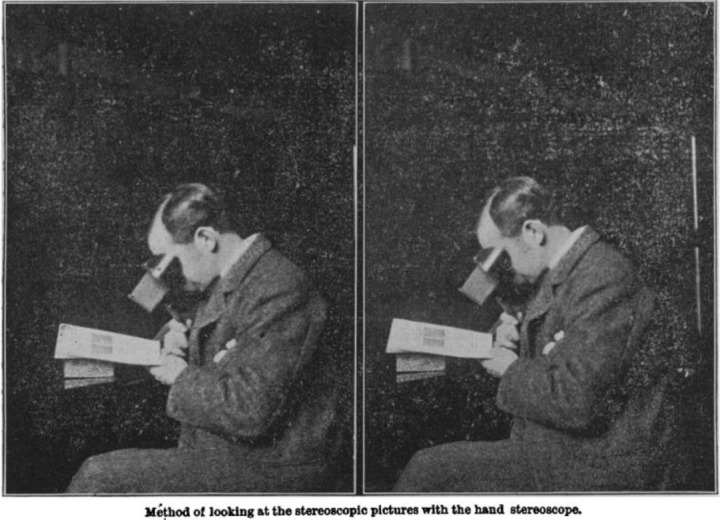
Stereoscope & user (BMJ). Image provided courtesy of the Robert L. Brown History of Medicine Collection, University at Buffalo Libraries.

The device known as the “stereoscope” was originally invented in 1838 by Sir Charles Wheatstone. In 1850, Sir William Brewster reconfigured the Wheatstone model to specifically enable its use as an image-viewing instrument [21]. The 3D effect of the stereographic image is produced as each eye views one of the double photographs, each taken at the same time from cameras 6 cm apart (the pupillary distance). When these photographs are viewed simultaneously through a stereoscope, the mind merges and projects a single seamless 3D image [21]. The optical novelty of the stereoscope resulted in, from 1858 to 1920, the proliferation of an estimated 3.5–4 million stereoview images for sale in the United States [21]. Major producers, such as Underwood and Underwood and the Keystone View Company, marketed a diverse range of stereographic image collections, including international travel sets, natural history, architecture, and footage of the First World War. In many ways, the stereoscope craze indicated the cultural importance of image production, circulation, and consumption and initiated a novel, affordable, and obtainable method of viewing the world (Figure 1).

At the turn of the twentieth century, the stereoscope became a valued tool of Western medicine, enhancing medical visualization and pedagogy and offering a striking visual departure for clinicians and students. A 1906 article published in the *American Journal of Surgery* touted the emerging pedagogical utility of the stereoscope: “Two means of realistic pictorial demonstration are creeping into the teaching of medicine—a science that avails itself so much of all the other arts and sciences. These means are the stereoscope and the vitascope (moving picture)” [22]. Likewise, as Jas. Mackenzie Davidson wrote in an 1898 issue of *The British Medical Journal,* “the advantages of stereoscopic photography for the purpose of recording and illustrating medical and scientific work is very great. For years past I have used it with the greatest benefit to myself and to students” [23]. The ascendency of stereoscopic visualization in the medical world resulted in the publication of highly detailed and oftentimes multivolume image collections, including *The Edinburgh Stereoscopic Atlas of Anatomy* (1905), *Imperial Stereoscopic Anatomy of the Head and Neck* (1909), and *The Stereoscopic Skin Clinic* (1911), among others [24–26].

**Figure 2:**
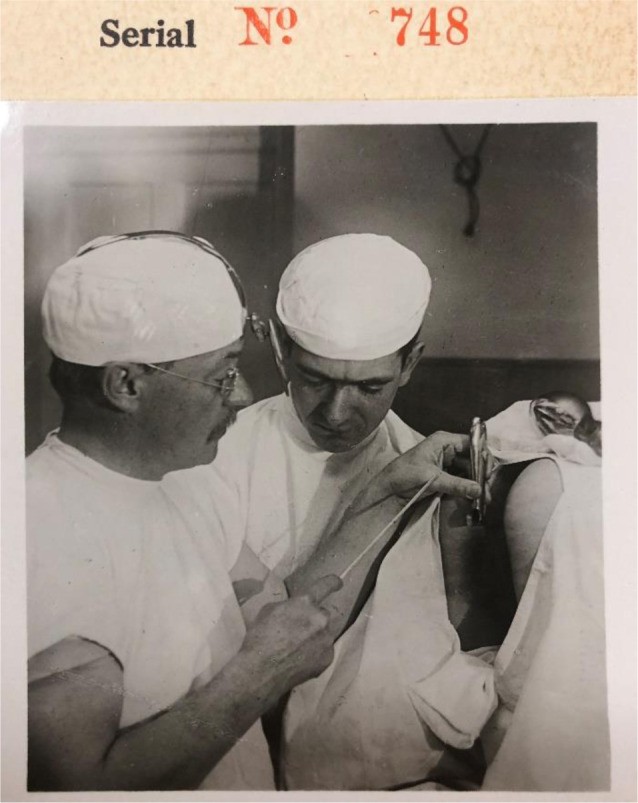
Dr. Howard A. Kelly (on the left). Image provided courtesy of the Robert L. Brown History of Medicine Collection, University at Buffalo Libraries.

One distinctly prolific contributor to what may be termed the “clinical stereoscope genre” was the American gynecologist Dr. Howard A. Kelly (1858–1943) (Figure 2). Counted among an elite group of physicians that established The Johns Hopkins Hospital in Baltimore, Maryland, Kelly was instrumental in creating the specialty of gynecology and known for his celebrated publications *Operative Gynecology* (1898) and *Biography of American Physicians* (1912) [27]. Kelly's oeuvre also includes his monumental forty-two-volume stereoscope project, *The Stereo Clinic.* These volumes were authored not only by Kelly, but also by other eminent contemporaries such as Drs. Charles Mayo, William Mayo, and George Crile.

## *THE STEREO CLINIC*'S PEDAGOGICAL & CLINICAL VISIONS

Kelly's *The Stereo Clinic* was a high-quality publication designed for student and professional use. Within a slipcase, each volume consists of vertically formatted pages, with single-sided printing, bound by two steel rings. The rings have narrow slits so that readers can remove an individual heavy-cardstock page to view the stereoscopic image. This two-ringed format was designed for flipping. Front and back hardcovers are lined with white, holographic fleur-de-lis-themed paper, and a coat of arms and Latin motto, *Sublimiora Spectemus* (“Let us look to higher things”), adorn the title page. Far from trivial production minutiae, these details suggest that Kelly viewed *The Stereo Clinic* not as a disposable textbook, but a tasteful and permanent addition to the user's library.

**Figure 3:**
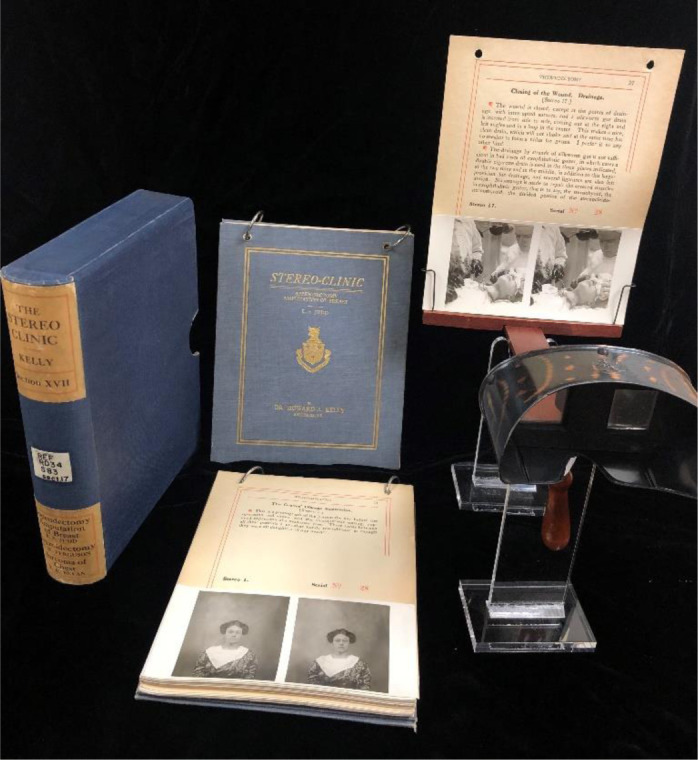
Image of *The Stereo Clinic* stereoscope, contents, pages, and slipcase. Image provided courtesy of the Robert L. Brown History of Medicine Collection, University at Buffalo Libraries.

Aside from the work's aesthetics, each of Kelly's volumes describes the step-by-step processes (ranging from five to forty-nine steps depending on complexity) of gynecologic and surgical procedures, from bladder examination and the catheterization of the uterus (v. II) to *Abdominal Pan-hysteromyomectomy in a Case of Double Uterus and Vagina* (v. IV) [9, 16]. Based on the twelve extant volumes available at the University at Buffalo's Robert L. Brown History of Medicine Collection, Kelly and his collaborators appear to have modelled and operated exclusively on live patients (as opposed to cadavers). In the style of a how- to manual, each page provides a written narrative, describing the steps of each surgical procedure, with a bottom half featuring a stereoview of Kelly or an invited surgeon performing the narrated activity (Figure 3).

Kelly understood *The Stereo Clinic* as an egalitarian education project whose goal was to transmit high-quality instructional content to both students and practicing surgeons working beyond the reach of ivory tower resources. Although ensconced in academia himself, Kelly was acutely aware that not all medical students and physicians had unhindered access to state-of-the-art educational tools, as many studied and worked far from urban teaching hospitals and well-stocked libraries. He notes on the dedication page of Volume IV:

This book is dedicated to my warm personal friend of N. A. Powell of Toronto because … he is the friend and champion of those for whom it is intended, physicians and students, scattered throughout his country and ours, who, lacking clinical facilities of great cities, are yet making a noble fight for the best things in medicine and history [16].

Thus, *The Stereo Clinic* may be first properly apprehended as a pedagogical bridge that connected the “scattered” members of the medical profession as well as an early form of continuing education for established physicians. The central concern seems to have been addressing the disparities in educational resource accessibility. In Kelly's 1943 obituary published in the *Transactions of the American Gynecological Society*, Guy Hunner, a former student and colleague, emphasized this egalitarian inspiration: “His passion for making operative procedures plain to the student, and especially to the rural surgeon who does not have easy access to large surgical clinics, led to the development of his *Stereo Clinics*” [28]. Exhibited here is the belief that *The Stereo Clinic* did indeed extend a first-rate educational tool to students and working professionals, even those on the periphery of the mainstream medical institution.

To realize this egalitarian vision, *The Stereo Clinic*'s first objective was to simulate the pedagogical experience of in-person surgical demonstrations while eliminating the quotidian inconveniences of the average classroom experience, such as crowded spaces and obstructed views. Seeking to achieve the synchronicity of a live clinic, Kelly's narrative voice uses vocal queues and directives that he would employ before a real audience. For example, in Volume VII, Kelly writes, “I would like to show today one of my methods of recording observations in tumor cases, displacements, or exudations” [15]. Kelly's metacommentary follows a typical classroom script, allowing him to connect with and guide his viewers through the surgical procedures depicted through the stereoscope. He also endeavored to reproduce the sensory experience of in-person demonstrations, writing that *The Stereo Clinic* was devised “so as to bring the observer *into a close and living touch* with each operation in process” [emphasis added] [9]. The stereoscope's 3D views enable users to study the patient and procedure in realistic physical depth and detail. Interestingly, by emphasizing the haptic and the visual, Kelly wanted stereoscope users to sense a felt proximity to the patient on the operating table. Indeed, just as consumers “traveled” the world in their armchairs using recreational stereoscopes, Kelly asks his users to imagine that they are seated in his clinic. Simulating real-life instruction underpins a key component of *The Stereo Clinic*'s educational value, that is, its ability to engage students as though they were experiencing live demonstrations.

A second important way that *The Stereo Clinic* realized Kelly's egalitarian vision was as a highly impactful educational tool. For Kelly, “the stereogram has an extraordinary value as a teaching agency” [9]. Aside from the stereoscope's practical conveniences, such as portability, it allowed users to access an enhanced plane of medical visualization that provided detailed 3D renderings of the patient's body and surgical procedures. Kelly regarded the stereoscope as a remedy to the perennial limitations of 2D medical drawings. “Surgeons,” writes Kelly, “have long been conscious of a want in the way of illustrating their operations not as yet perfectly met by flat illustrations however admirably done” [9]. He also nods to fine anatomical plaster works, but his egalitarian conscience recognizes that these “specimens are not available for all, and apart from their being stored in a laboratory, they are in the gross bulky as well as fragile” [9]. The stereoscope therefore “meets the want and offers an ideal solution of this question” [9]. As opposed to the flatness of illustrations and the inaccessibility of models, in “a stereoscopic picture (a stereogram) of a surgical operation, we have perfect natural relief, and an untouched presentation of the subject, free from all error or bias of interpretation” [9].

In addition to improving medical visualization, Kelly's *The Stereo Clinic* offered other salient pedagogical advantages. First, the images are tutorial in nature, allowing users to return to and study certain steps of the depicted surgical procedure if clarification or additional perspective is required. The pedagogical value is clear: “The steps of an operation not fully grasped, the observer at the stereo-clinic simply goes back and reviews, and so can make sure that he is following accurately the technique of the procedure” [9]. *The Stereo Clinic* gave viewers what thronged dissection theatres could not—the ability to retrospectively “pause” and study a specific technique, corporeal angle, or description that was lost in a hectic moment of live observation. Kelly emphasizes that the stereogram “offers in addition the advantage of a prolonged and deliberate study of any given step in an operation, an advantage which no operator can often afford his visitors upon the living patient” [9].

## PROFESSIONAL & POPULAR RECEPTION OF *THE STEREO CLINIC*

The marked importance of Kelly's *The Stereo Clinic* was amply reflected in its professional and popular reception. Multiple points of consensus among the period's critics emerge, specifically on *The Stereo Clinic*'s pedagogical value, comprehension, convenience and user friendliness, visual precision, and high-quality presentation.

Overall, reception reflects the American medical community's esteem for Kelly's stereographic work. The *New York Medical Journal* noted that Kelly's “handsome volumes” were an educational tool that allowed users to view “in consecutive steps the whole operation, instead of following an operation quickly performed in a crowded amphitheater” [29]. Likewise, the *Journal of the American Medical Association* emphasized the stereoscope's simulation of real-life procedures: “Instead of trying to catch a hurried glimpse of a rapidly performed operation, one may sit down and view the various steps with almost the realism of the operating room” [30]. Elsewhere, an approving review published in the *American Journal of Surgery* quoted Kelly himself (originally published in Volume II of *The Stereo Clinic*):

In some respects, paradoxical and heretical as the statement may sound, an ideal stereogram is really better than a regular clinic … A stereogram offers in addition the advantage of a prolonged and deliberate study of any given step in an operation, an advantage which no operator can often afford his visitors upon the living patient [31].

A review printed in the 1911 issue of *The Journal of Osteopathy* agreed, lamenting “how unsatisfactory it is to sit in a hot operating pit for several hours” [32]. Kelly's *The Stereo Clinic*, however, allows the clinician “desiring to study an operation, gynecological or surgical, [to] sit down in his office at his leisure, the field of operation constantly before him, at close range, each step being explained more fully than it can possibly be done by a surgeon while operating” [32]. Indeed, the foremost value indicated in the reception literature was that *The Stereo Clinic* rectified, in part, the inadequacies of formal instructional exhibitions (those belonging to the realm of crowded classrooms, clinics, and surgical suites) by providing a realistic pedagogical alternative that could be pursued, without distraction, in one's library chair.

Aside from these advantages, there was general acclaim for the stereoscope's visual accuracy. The *American Journal of Clinical Medicine* remarked enthusiastically that Kelly's “stereoptical views” were “superior both to photographic reproductions and to plaster casts” [33]. Similarly, the *Journal of the American Medical Association* noted the triumph of the stereographic image over “the ordinary illustration, however well the latter may be executed” [30]. “Flat illustrations,” wrote one critic in the *American Journal of Surgery*, “cannot always give a complete notion of depth-relations; they fail to show structures in the same relief as seen at the operation itself” [31]. From this stance, *The Stereo Clinic* was seen as moving beyond the limitations of older modes of medical visualization to improve student education.

Reception was, however, not without critique. The *American Journal of Surgery* observed that “although the photographs are admirable and well mounted, and the relief effect is all that it should be, the structures appear cadaveric or, more accurately, as though of clay—they fail to give the impression of living tissue” [31]. That Kelly's examples lacked vivid authenticity was not viewed as being detrimental to the quality of the collection. This critical viewpoint, if anything, suggests that there might have been slight reluctance in the medical community to concede that ideal imaging could be fully achieved through the stereoscope, that is, that any technology could replace the human experience of in-person medical observation.

The *Journal of the Indiana State Medical Association* looked askance at the sanguinary messiness of the depicted surgical scenes:

One cannot but wish that the operator had either worn gloves or that his finger-nails had been cropped sufficiently short to prevent their having collected the blood which, appearing black as it does, gives to the student a very unpleasant sensation as to cleanliness [34].

The concern here was that the fingernails would impart to impressionable viewers an overall sense of unhygienic surgery.

Discussions on Kelly's *The Stereo Clinic* were not confined to professional medical circles. In 1912, the *Boston Evening Transcript* enthusiastically reported, “These pictured operations are designed for the use of those who have not time to attend clinics, or who may not have the opportunity of seeing noted surgeons at work” [35]. The same newspaper advertised in 1915 that *The Stereo Clinic* was displayed at Boston's Copley-Plaza, presumably for a medical conference or public viewing: “Dr. Kelly,” writes the journalist, “has staged or ‘*dramatized*' some eighty major operations by prominent surgeons” [emphasis added]. Interestingly, the stereoscope project was described as “something like a *moving-picture*” that appealed to “the surgeon who wants to brush up” or the medical student who “may sit comfortably at home and see as many clinics, all reproduced life-size, as he has sets of views” [emphasis added] [36]. As demonstrated, Kelly's *The Stereo Clinic* garnered public attention and was abundantly promoted. On the one hand, journal reviewers emphasized the stereoscope's practical conveniences and significant educational value. On the other hand, reviewers underscored the cinematic-like quality of Kelly's images, which speaks to the engrossing vividness of his individual slides.

## DISCUSSION

Even as professional and popular reception lauded the ingenuity of *The Stereo Clinic*, Kelly himself was not oblivious to the work's few shortcomings. He knew well that the stereoscopic images “at once stand out sharp and clear, and are perfectly adapted to teach the particular operative procedure for which they were taken” [9]. Yet, some images, however useful they were, did not capture the granular nuances of some surgical moments. For example, he notes that “bleeding tissues are difficult to photograph clearly” [9]. Furthermore, Kelly wrote: “much that I wanted to show my audience lay concealed in the dark cavity of the vagina or of the abdomen” [9]. In examining representative images, the darkness described by Kelly is at times conspicuous. Viewers can observe instruments receding into patients' bodies but, due to shading, cannot always discern the ideal positioning or anatomic landmarks (see v. XI) [19]. Shadows, blood, and obscured orifices were the drawbacks of black and white photographic imaging. These various imperfections Kelly readily admitted, but they did not thwart the egalitarian vision of *The Stereo Clinic*.

Today, 3D and VR technologies continue to permeate health science curricula. As librarians evaluate and promote such important technologies, we suggest close examination of issues related to their cost and access. In the spirit of *The Stereo Clinic,* both developers and champions of 3D technologies must consider general accessibility and determine how to best ensure that ***all*** students and practicing clinicians benefit from these exciting and impactful tools.
